# Pilot projects empower district supply chain management staff to strengthen health services in Indonesia

**DOI:** 10.1186/2052-3211-7-S1-P15

**Published:** 2014-12-17

**Authors:** Setiawan Suparan, Ketut Adnyana, Ivan Surya Pradipta, Nani Sukasediati, Prihatiwi Setiati

**Affiliations:** 1 USAID | DELIVER PROJECT, Jarkata, Indonesia; 2 School of Pharrmacy, ITB/Bandung Institute of Technology, Jawa Barat, Indonesia; 3 Faculty of Pharmacy University of Padjadjaran Bandung Unpad, Jawa Barat, Indonesia; 4 WHO, Jakarta, Indonesia; 5 Ministry of Health, Jakarta, Indonesia

## Background

Indonesia comprises 17,000 islands and 495 districts. Decentralization mandates district health services, but ensuring proper supply chain management (SCM) capacity at the district level is a challenge. The ministry of health developed and implemented SCM training modules and guidelines; however, weaknesses in the system remain, particularly related to human resources. The ministry in collaboration with universities and funders, piloted two projects that empower SCM staff. It will use the results to strengthen SCM at the district level.

## Method

Two pilot projects, with different timeframes and funding, focused on staff empowerment, local ownership, team work, local problem solving, and enhanced professionalism. The pilots were built around a standard SCM cycle and used self-assessments to identify gaps in the SCM system. The approach also facilitated team work to develop and implement a corrective action plan.

## Results

The pilot sites exhibited the same gaps. Which included: limited human resource capacity, and a lack of appropriate standard operating procedures (SOPs). A three-month internship for newly graduated pharmacists and pharmacy students, with SCM skills, was used to strengthen HR capacity. SCM training conducted by the interns for district SCM personnel proved effective, improving staff performance. Other interventions included reviewing and revising both SOPs and a quality assurance check list for SCM. Collaboration among SCM staff and managers was intensified, using an Integrated Drug Management approach. All the pilot sites now use standard SCM SOPs and have an Integrated Drug Management team.

## Discussion

The internship program was very effective in increasing SCM performance and pharmacy services at the district level. We confirmed that SOPs are critical tools that should be used to facilitate standard, good quality performance. Empowering district personnel to review their own SCM program in a systematic manner and to prepare a follow-on action plan proved highly successful; this approach will now be used in expanded efforts to improve the district level SCM system and staff capacity.

## Lessons learned

Empowering SCM staff through local ownership and self-assessment is an effective and sustainable way to create SCM interventions tailored to district needs. It also builds district staff commitment and confidence. Newly graduated pharmacists with SCM skills can act as change agents for improving SCM.

